# Integrating Mobile Text Messaging Pre-Exposure Prophylaxis Navigation Services Into a Home HIV and Sexually Transmitted Infection Self-Testing Program in the United States: Formative Work and Pilot Implementation Study

**DOI:** 10.2196/83733

**Published:** 2026-04-21

**Authors:** Albert Liu, Cat-Dancing Alleyne, Juwann Moss, Janie Vinson, Kyle Lafferty, Sarah Quintana, Nancy Duenez, Kaitlin Lopez, Deejay Johannessen, Anthony Fraide, Susan Buchbinder, Hyman Scott, David Glidden, Jen Hecht

**Affiliations:** 1 Bridge HIV, San Francisco Department of Public Health, 25 Van Ness Avenue, Suite 100, San Francisco, CA, 94102, United States, 1 628-217-7408; 2 Department of Medicine, University of California San Francisco, San Francisco, CA, United States; 3 Sacramento County Public Health, Sacramento, CA, United States; 4 Tarrant County HIV Administrative Agency, Fort Worth, TX, United States; 5 HELP Center for LGBT Health and Wellness, Fort Worth, TX, United States; 6 Department of Epidemiology and Biostatistics, University of California, San Francisco, San Francisco, CA, United States; 7 Springboard HealthLab/BHOC, Richmond, CA, United States

**Keywords:** pre-exposure prophylaxis, PrEP, HIV testing, mobile health, mHealth, HIV prevention, HIV, navigation, technology, formative, development, mobile technology, text-messaging, SMS text messaging, self-test

## Abstract

**Background:**

HIV testing is the gateway to the HIV prevention continuum and offers an important opportunity to provide HIV prevention services. TakeMeHome.org is an online program that enables state and local health departments to offer free in-home HIV and sexually transmitted infection self-testing. As few TakeMeHome users have used pre-exposure prophylaxis (PrEP), there is an opportunity to link TakeMeHome users to PrEP information and services.

**Objective:**

The aim of this study is to develop an implementation strategy to link HIV or sexually transmitted infection self-testers from online orders to PrEP services via direct digital linkage to a novel SMS text messaging navigation program.

**Methods:**

PrEPmate is an evidence-based bidirectional text-messaging platform that has demonstrated increased PrEP retention and adherence. We developed a novel program to link TakeMeHome testers to mobile SMS text messaging PrEP navigation via PrEPmate. We conducted focus groups among TakeMeHome users to elicit preferences for linkage from TakeMeHome to PrEPmate. Based on these focus groups, we revised the content and functionality of this linkage intervention. In October 2023, we launched a pilot implementation study in 2 US Ending the HIV Epidemic jurisdictions: Sacramento, California, and Tarrant, Texas.

**Results:**

Thirteen TakeMeHome users participated in 4 focus groups (mean age 31.5 years; n=4, 31% Latinx, n=2, 15% Black; n=9, 69% never used PrEP). When shown wireframes of the TakeMeHome or PrEPmate linkage, most thought they were easy to navigate and user-friendly. They liked the privacy of connecting with a PrEP navigator using SMS text messaging. Participants recommended providing a clear description of PrEP and PrEPmate services and indicating that PrEP is low or no cost on the TakeMeHome website. On the PrEPmate landing page, they recommended adding language on confidentiality and the partnership with TakeMeHome to show that both services are connected. Once enrolled, they recommended weekly or biweekly check-ins to assist with PrEP navigation. Overall, 92% (12/13) of focus group participants were likely to use PrEPmate to learn more about PrEP and/or link to PrEP services. From October 2023 to May 2024, among 537 individuals who ordered test kits and were not on PrEP, 169 (31%) were linked to the PrEPmate page, and 86 (16%) enrolled in PrEPmate. PrEP navigation was provided via SMS text messaging or phone, with 46 (53%) receiving PrEP education and 26 (30%) in various stages of starting PrEP. In exit interviews, participants found the intervention easy to use and appreciated being connected with an experienced PrEP navigator who helped them access PrEP.

**Conclusions:**

Through user-centered design, we successfully developed a program to link TakeMeHome testers to PrEP navigation via PrEPmate, with high feasibility and acceptability of the intervention and a substantial number of clients starting PrEP. The next steps will involve evaluating the effectiveness of this program on a larger scale and, if successful, expanding PrEPmate navigation to all Ending the HIV Epidemic jurisdictions using TakeMeHome.

## Introduction

HIV testing is the gateway to the HIV prevention and care continua, enabling timely diagnosis, linkage to antiretroviral therapy and HIV care, and offering effective HIV prevention strategies, including pre-exposure prophylaxis (PrEP) [1]. Despite the Centers for Disease Control and Prevention (CDC) recommendation that all persons aged 13 to 64 years receive an HIV test [2], testing coverage remains suboptimal, with only 36% of adults younger than 65 years in the United States having ever been tested for HIV, and lower testing rates among adolescents and young adults [3,4]. Web-based and mobile intervention models of HIV testing have demonstrated promise in reaching populations at risk for HIV and sexually transmitted infections (STIs), including those never tested and those in high HIV seroincidence populations, the priority population for rollout of PrEP [5,6]. Digital platforms can provide tailored education, testing reminders, testing locators, and home delivery of HIV and STI test kits [7].

Developed by Building Healthy Online Communities (BHOC) and in collaboration with the National Alliance of State and Territorial AIDS Directors and Emory University as a public-private partnership, TakeMeHome.org is an online program enabling state and local health departments to offer free in-home HIV and STI testing [8]. TakeMeHome has partnerships with several dating apps to provide in-kind links to resources and free messaging. Launched in March 2020, the TakeMeHome program has distributed more than 70,000 HIV self-testing kits in 29 jurisdictions across 20 states, with 31% of testers reporting testing for the first time [9]. In 2023, a national version of this program, Together TakeMeHome, was launched, and in the initial year’s rollout of online access to HIV testing, of nearly 220,000 persons requesting test kits, 55% were persons from the CDC’s priority populations, 24% had never had an HIV test, and another 25% had not tested in the last year [10].

While linkage to HIV care and antiretroviral therapy is generally high after HIV self-testing, rates of linkage to HIV prevention services in this setting remain low. In a recent systematic review and meta-analysis, rates of confirmatory testing, ART initiation, and HIV care were 92%, 89%, and 84%, respectively, after a reactive HIV self-test; however, linkage to PrEP after a negative HIV self-test result was low at 9%, with even lower rates (4%) observed among key populations [11]. Similarly, while TakeMeHome.org provides a novel national strategy to increase HIV and STI testing among priority populations in the United States, there is a missed opportunity to engage these individuals in PrEP services. Only 8% of TakeMeHome users and 7% of Together TakeMeHome users were currently on PrEP [9,10]. Multilevel barriers to PrEP uptake include lack of awareness of PrEP, stigma and discrimination, medical mistrust, concerns about side effects and/or PrEP effectiveness, limited access to PrEP providers, transportation barriers, and the cost and complexity of insurance coverage [12-14]. As prior digital HIV prevention tools have relied on a more passive referral of clients to PrEP services, a digital health strategy that integrates self-testing with direct PrEP navigation and linkage via SMS text messaging is a novel approach that could help address a number of these barriers and increase PrEP uptake among priority populations using home HIV and STI testing services. In this study, we conducted qualitative formative work to develop a novel program for linking TakeMeHome testers to mobile SMS text messaging PrEP navigation via PrEPmate, a CDC-endorsed bidirectional text-messaging platform that previously increased PrEP retention in care and adherence among young sexual minority men and has subsequently been tailored for transgender and Spanish-speaking populations [15]. PrEPmate was originally designed to provide weekly check-in messages to facilitate communication between the clinic and patients, help triage those who need additional assistance, and provide daily pill reminders for taking PrEP. We used a user-centered design approach in which end users are meaningfully involved in application development to ensure that our tools are functional, usable, and customized to meet their needs [14,16]. We then assessed the feasibility, acceptability, and preliminary effectiveness of this intervention to increase PrEP uptake among TakeMeHome users in Sacramento, California, and Tarrant County, Texas, both Ending the HIV Epidemic (EHE) priority jurisdictions in the United States with a large volume of HIV and STI testing via TakeMeHome and interest and capacity to participate. This implementation study is focused on the stages of developing or adapting a solution and piloting and evidence generation.

## Methods

### Study Objectives

The main objectives of this study and the overall aim of the implementation were to develop and refine the TakeMeHome to PrEPmate linkage program via focus groups and evaluate the feasibility, acceptability, and preliminary effectiveness of this novel approach via a pilot implementation study. The target population for this program includes individuals testing via the TakeMeHome.org program who are not taking PrEP but could benefit from it.

### Focus Groups for Developing the TakeMeHome to PrEPmate Linkage

We conducted 4 focus groups with TakeMeHome users recruited from Sacramento, CA, and Tarrant County, TX. Eligible participants for the focus groups were individuals who had used the TakeMeHome.org testing service within the past 12 months in the 2 participating jurisdictions, were HIV-uninfected by self-report, and were currently or previously on PrEP or interested in starting or learning more about PrEP. Recruitment included sending an Institutional Review Board–approved recruitment email asking individuals if they would be interested in participating in a 90-minute focus group conducted virtually, with a link to complete a secure contact information form. A convenience sample of eligible participants was consented online using an information sheet prior to the initiation of the focus group. Focus groups were conducted using a Health Insurance Portability and Accountability Act–compliant videoconferencing program (Zoom). Using a discussion guide, we elicited participants’ experiences with accessing PrEP; gathered feedback on questions to be added to TakeMeHome.org to identify individuals not currently on PrEP but interested in learning more about this prevention approach; reviewed PrEP educational materials to be incorporated into TakeMeHome.org; explored the optimal strategy to facilitate successful linkage between TakeMeHome.org and PrEPmate; and assessed preferences for PrEP navigation and initiation within PrEPmate. Participants were shown wireframes of the TakeMeHome and PrEPmate screens and provided feedback on these images. Upon completion of the focus group, participants completed a brief online survey via Qualtrics to collect sociodemographic information and assess potential interest in PrEPmate. Focus groups were led by 2 research staff members trained in qualitative methods and user-centered design principles and fluent in English or Spanish. Groups lasted 90 minutes and were audio-recorded and professionally transcribed and translated (for Spanish focus groups) verbatim.

Guided by user-centered design principles [17], we used rapid qualitative methods to analyze the focus group data [18]. After each focus group, key findings from the discussion were summarized in a debrief note drafted and reviewed by 2 research team members who attended the group. Data captured in this template were based on the interview guide, agreed upon via consensus of the investigator team, and focused on information needed to develop the linkage between TakeMeHome and PrEPmate. Upon receipt of transcripts from the focus groups, members of the team reviewed the transcripts and captured additional details to ensure completeness of the debrief reports. These data were used to refine this linkage before the initiation of the pilot study. For publication of this manuscript, we developed a coding matrix based on the interview and debriefing guides that was agreed upon by the research team. Coding was conducted by 2 members of the research team who reviewed each other’s work and came to consensus with input from the research team. Relevant quotes were extracted from the primary transcripts for each of the codes.

### Development of the TakeMeHome to PrEPmate Linkage Intervention and Description of the Intervention

With input from the qualitative data, BHOC developers built out the new flow and added screens prompting users who were not on PrEP to learn more and, if interested, click to get connected to the PrEPmate landing page, which provided information about the program and allowed users to sign up for the service. After enrolling in the program, a PrEP navigator reached out to the client and provided text- or phone-based support to answer questions and facilitate access to PrEP. The system was designed so that all TakeMeHome users indicating interest in PrEP would be offered the linkage to PrEPmate during the pilot study period. PrEPmate is a cloud-based, software-as-a-service solution [19] hosted on Health Insurance Portability and Accountability Act–compliant servers that meet global standards for privacy, security, and redundancy, and a business associate agreement has been established to ensure confidentiality and privacy of data. Development of this linkage intervention was funded via a Gilead Sciences NOVA (Novel Research to Advance HIV Prevention) grant provided to the Bridge HIV research team at the San Francisco Department of Public Health. The budget for this study covered the costs of conducting focus groups and the pilot study, technical development of the TakeMeHome or PrEPmate linkage by BHOC, and recruiting participants into the study. BHOC (developer of TakeMeHome) and WelTel (which supports the PrEPmate platform) are both sustainable programs funded through government and public health grants, pharmaceutical and foundation funding, and other partnerships. TakeMeHome’s business model is based on health departments’ and community-based organizations’ funding covering the cost of the kits, shipping, and basic site operations for their constituents, which are identified by geographic area.

### Pilot Study to Assess the Feasibility and Acceptability of the TakeMeHome to PrEPmate Linkage

After finalizing the TakeMeHome to PrEPmate linkage, we assessed the feasibility and acceptability of this novel PrEP uptake strategy in a pilot study conducted during a 9-month period (October 2023 to May 2024). Individuals linked to PrEPmate via TakeMeHome.org as part of standard-of-care services were sent an Institutional Review Board–approved recruitment text message invitation approximately 1 month after enrolling in PrEPmate to participate in a 10 to 15 minutes study survey and a 60 to 90 minutes exit interview (among a subset of participants) to evaluate the PrEPmate linkage and navigation process. Interested participants were consented online using an information sheet and directed to complete the online survey. The survey collected information about participant sociodemographics, mobile phone ownership, prior PrEP knowledge and use, and whether they were successfully linked to PrEP services. Acceptability and patient satisfaction were assessed using the System Usability Scale (SUS) and Client Satisfaction Questionnaire-8 (CSQ-8), 2 validated tools assessing usability and acceptability with demonstrated high internal consistency across several studies [20,21]. The SUS is a 10-item scale that measures a system’s usability on a scale of 0 to 100, with a score greater than 80.3 consistent with an “excellent” rating and scores 68 to 80.3 consistent with a “good rating” [22]. The CSQ-8 is an 8-item scale that measures client satisfaction on a scale of 8 to 32, with scores above 26 considered high [23]. We compared sociodemographics of PrEPmate survey completers with available data from overall TakeMeHome users from the 2 jurisdictions during the same time period using Fisher exact test.

We also conducted exit interviews with randomly selected participants who completed a survey to elicit feedback on their experience being linked from TakeMeHome.org to PrEPmate and receiving telenavigation services via PrEPmate and any recommendations to improve their experience with these services. Exit interviews were conducted remotely via Zoom using a semistructured interview guide. Interviews lasted 60 to 90 minutes and were audio-recorded and transcribed. Exit interviews and text message content were analyzed using methods similar to those described for the focus groups above.

Feasibility was assessed descriptively via deidentified data in reports generated from TakeMeHome.org and PrEPmate, including monthly counts of the number of kit orders per month, the number of individuals not on PrEP, the number of individuals who clicked over from TakeMeHome to the PrEPmate landing page, and the number who enrolled in PrEPmate. PrEP outcomes (those in the process of starting or who had started PrEP) were assessed via navigator reviews of clinic records.

### Ethical Considerations

All study procedures were approved by the University of California San Francisco Institutional Review Board (UCSF IRB 22-37766). Focus group participants were provided with an information sheet in English or Spanish describing study procedures and the risks and benefits of study participation. Pilot study participants were provided with an information sheet describing the study survey and exit interview procedures. Focus group transcripts were transcribed in a way that removed any identifying information. Survey data for both phases contained only deidentified data and were coded by a subject number. A business associate agreement was established with both developers to ensure confidentiality and security of data. Prior to enrolling in PrEPmate, participants were provided a data privacy policy statement explaining the use and safe storage of their data. Data collected via PrEPmate are stored in Health Insurance Portability and Accountability Act–compliant cloud-based servers and are owned by the research team at the San Francisco Department of Public Health. Focus group participants were paid US $75 for completing the focus group; pilot participants were paid US $70 for completing the survey and US $120 for completing the exit interview. As the use of TakeMeHome and PrEPmate was considered part of the standard of care, participants were not paid for signing up for these services, but only for the completion of the study survey and/or interview.

## Results

### Findings From Focus Groups

#### Participant Characteristics

From March to May 2023, we contacted 29 TakeMeHome users, of whom 7 were ineligible and conducted 4 focus groups with 13 TakeMeHome users (response rate 65%), with 7 (54%) participants from California, 5 (38%) from Texas, and 1 (8%) from another state. The median age was 31 years; 4 (31%) identified as Latinx, 2 (15%) Black, 6 (39%) white, and 2 (14%) Asian. Overall, 11 (85%) were cisgender male, 1 (8%) genderqueer, and 1 (8%) cisgender female; 11 (85%) were insured and 9 (69%) had a primary care provider; 2 (15%) were currently on PrEP, 2 (15%) had previously used PrEP, and 9 (69%) had never used PrEP.

#### Privacy and Confidentiality and Ease of Use as Motivators for Engagement

Participants in Texas reported stigma around PrEP and being part of the gay community as barriers to accessing PrEP services. When asked about barriers to getting on PrEP, one participant in Texas said,

*I’d say the stigma. You don’t want to be like the gay person that’s going to get the HIV drugs and all the things that come with that…I live in Ft. Worth now…it’s just if you go to the wrong place, you’re going to be looked at the wrong way, and it’s a little intimidating sometimes.*

**Figure 1.**
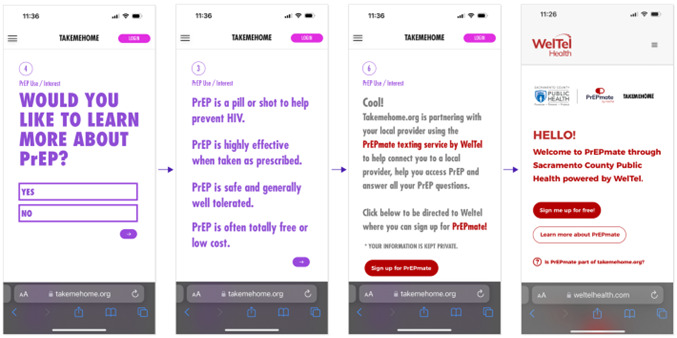
Screenshots of TakeMeHome to PrEPmate linkage pages. PrEP: pre-exposure prophylaxis.

When shown wireframes of linkage pages (Figure 1), most thought they were easy to navigate and user-friendly, and they liked the privacy of connecting with a PrEP navigator using SMS text messaging. One participant in Texas said,

*I think it’s very easy to navigate. If I were on this website and I were trying to find PrEP, I would definitely feel like it was very user friendly and very easy to navigate, just going through it. You know, I like that you can sign up for, like, a text, text communication. Because, I mean, everybody nowadays texts.*

#### Need for a Clear Description of PrEPmate and Its Connection to TakeMeHome

On the TakeMeHome PrEP page, participants recommended a clear description of PrEPmate services, with 1 participant in Texas saying,

*I guess just informing that there is this text-based service where you can chat with live navigators to ask your questions in a completely confidential manner. Just knowledge that there are actual live experts on the subject that you can access through the privacy of text message at your convenience 24/7, to ask literally any question you can think of about this.*

On the PrEPmate landing page, participants recommended adding language on confidentiality and the partnership with TakeMeHome to show both services are connected. One participant in Texas remarked,

*And also having on the website, maybe like, “Supported by TakeMeHome.” So, there’s that connection. And there’s another trusted organization. So, it’s like if that organization’s supporting that, and I’m trusting that, then it’s probably legitimate.*

Similarly, another participant said,

*Making a more direct link to TakeMeHome, as a service that you’re already familiar with, and then PrEPmate. So, kind of bridging that gap between the relationship.*

#### Need for Clear Cost and Insurance Information for PrEP

Participants agreed that it would be important to have information about the cost of PrEP and whether it can be accessed at low or no cost. One participant in Texas noted,

I think, for me, medication, if the costs, it kind of scared you away without insurance.

Another participant in Texas said,

*Cost is the big thing. So, if we put here that, you know, PrEP is usually free or low cost, it would be more enticing to people to go ahead and learn more and continue on with this process.*

#### Desire for Immediate Human Connection (Live Navigator)

Several participants highlighted the importance of speaking with a live person knowledgeable about HIV prevention to answer their questions about PrEP. One participant in Texas said,

*I definitely feel like having someone, either, like, a live person or chat feature… having that person who’s very knowledgeable—someone who’s professional, someone who knows about it—to be able to chat with. I feel like that is something that, you know, I really enjoy. You know, getting that information and getting all my questions answered, more importantly.*

They also suggested minimizing the number of questions prior to enrolling in PrEPmate, as this may contribute to drop-off, and 1 participant even suggested providing a direct link to PrEPmate first, and then having PrEP navigators ask the triage questions. A participant in Texas said,

*It seems to me like you would want to funnel everybody directly into the text service, period. And then once you got them, and you got them in the text service, then you ask them the triage questions through the text service.*

#### Suggestions for Follow-Up Frequency, Language and Tone of Messages, and Laboratory Reminders

For ongoing navigation, participants recommended weekly or biweekly check-ins to assist with PrEP navigation. They recommended follow-up texts using casual language, with an example being

*Hey, I haven’t heard from you in a while. You’ve got whatever navigators here that can answer all your questions in private. Just reach out anytime if you have any questions. We’re here to help.*

One participant commented on the challenges with completing blood work for PrEP follow-up visits and said it would be helpful if PrEP navigators could send text message reminders regarding when labs are due and help make those appointments. One participant in Texas said,

*I think that would be a great idea. Because that’s part of my problem, is time flies by and then I won’t get a refill and it’s because I didn’t get my labs done. And now it’s like, oh, everything comes to a stop. But if I had maybe text reminders, like, “Hey, your labs are due in 2 weeks.” “Hey, your labs are due in a week,” that would really help me not get in those really bad binds, for sure.*

In the postinterview survey, 92% (12/13) of participants reported they were likely to use PrEPmate to learn more about PrEP and/or link to PrEP services. Based on these findings, refinements were made to the TakeMeHome and PrEPmate sites prior to testing in the pilot study (Textbox 1).

Textbox 1. **Refinements made to TakeMeHome and PrEPmate based on feedback from focus groups.**

**TakeMeHome**

- Added clear description of PrEPmate services
- Added information that PrEP is available at low or no cost
- Reduced number of screens required to be completed prior to linkage to PrEPmate
- Incorporated colors of the PrEPmate page (red font) into the final screen to promote consistency

**PrEPmate**

- Added logos of TakeMeHome, local health department or clinic, and PrEPmate, and a description of how sites are related
- Added information about privacy and confidentiality, including a link to the PrEPmate privacy policy
- Reduced number of questions and data elements collected as part of onboarding
- Used personalized, conversational language in initial welcome text messages to clients

### Findings From Pilot Study

From October 2023 to May 2024, we conducted a pilot of the TakeMeHome or PrEPmate linkage in Tarrant County, Texas, and Sacramento, California, 2 EHE jurisdictions using TakeMeHome. Overall, 86 individuals enrolled in PrEPmate, and 31 (36%) completed the follow-up study survey.

**Figure 2.**
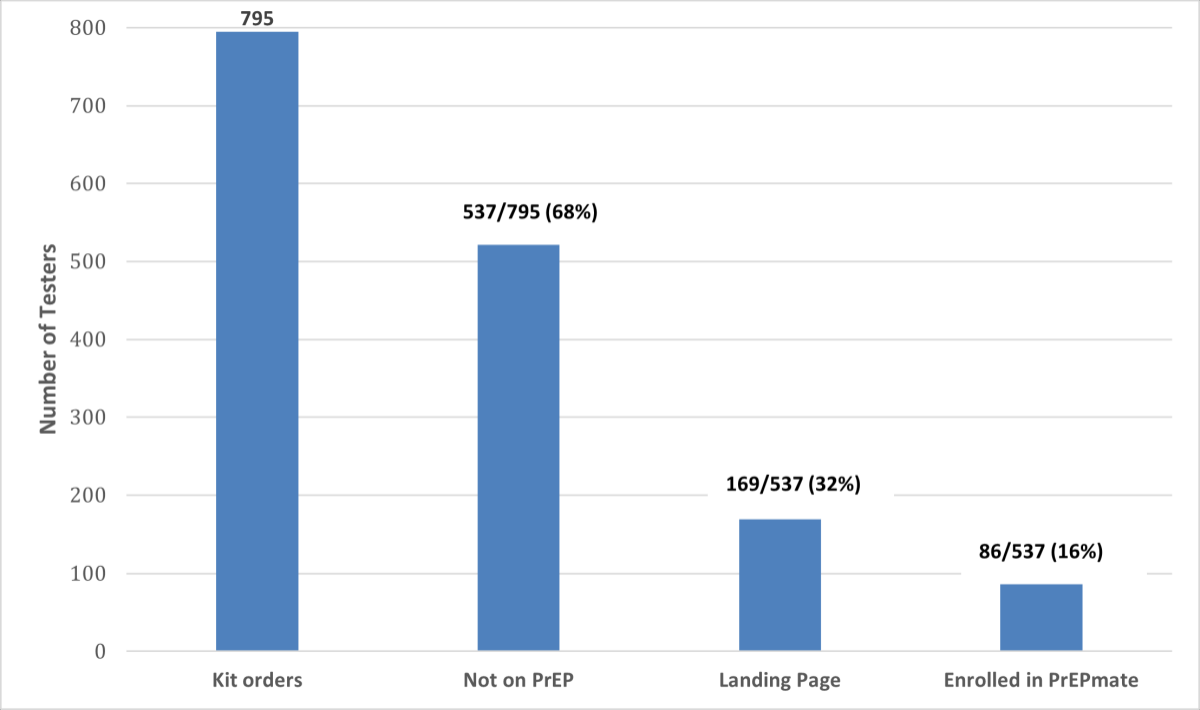
TakeMeHome to PrEPmate cascade. PrEP: pre-exposure prophylaxis.

**Figure 3.**
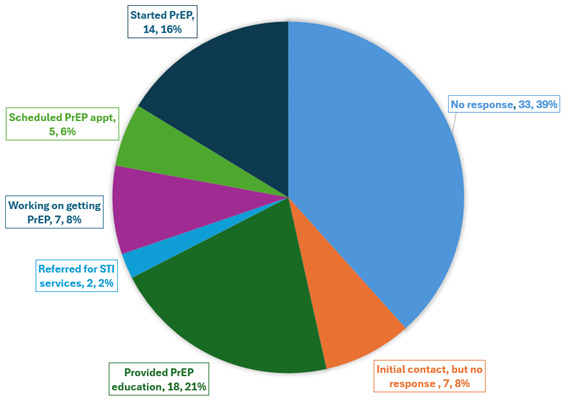
Pre-exposure prophylaxis (PrEP) outcomes for individuals enrolled in PrEPmate (N=86). STI: sexually transmitted infection.

The TakeMeHome to PrEPmate cascade by month is shown in Figure 2. Among 537 individuals who ordered test kits and were not on PrEP, 169 (31%) were linked to the PrEPmate page, and 86 (16%) enrolled in PrEPmate (median age 30 years, 402, 75% were interested in PrEP). PrEP navigation was provided via a local navigator through the PrEPmate platform. Among those enrolled, 46 (53%) were provided PrEP education, 7 (8%) were working on getting on PrEP, 5 (6%) had a scheduled PrEP appointment, and 14 (16%) had started PrEP (Figure 3).

Demographic characteristics of the 31 participants who completed the 1-month survey are shown in Table 1. The median age was 34 years; 22 (71%) participants were from California; and 17 (55%) identified as a man, 11 (35%) as a woman, and 3 (10%) as a transgender woman. Approximately one-third (10, 32%) identified as Hispanic/Latino/a/x, and 5 (16%) African American. The median number of sex partners in the last 3 months was 3. Overall, 25 (81%) had health insurance and 17 (55%) had a primary care provider; 20 (65%) had previously heard of PrEP, and 7 (23%) had previously used PrEP (but not currently). Participants who enrolled in PrEPmate and completed the 1-month survey were similar to overall TakeMeHome testers from the 2 jurisdictions with respect to age, race and ethnicity, gender identity, and reported sexual activity (see Multimedia Appendix 1). Among the 31 survey participants, 10 (32%) started PrEP as a result of PrEPmate, and an additional 14 (45%) were in the process of getting on PrEP. Among the 21 who had not yet started PrEP, most 16 (75%) were very or somewhat interested in taking PrEP.

**Table 1.** Sociodemographic characteristics, pre-exposure prophylaxis interest, and uptake among focus group and survey participants in Sacramento, California, and Tarrant County, Texas.

Characteristic	Focus group (N=13)	Survey (N=31)
Age (y), median (IQR)	31 (24-37)	33.7 (25-41)
Location, n (%)		
California	7 (54)	22 (71)
Texas	5 (38)	9 (29)
Another state (Oregon)	1 (8)	0 (0)
Gender identity, n (%)		
Man	11 (85)	17 (55)
Woman	1 (8)	11 (35)
Transgender woman/genderqueer	1 (8)	3 (10)
Sexual orientation, n (%)		
Gay	—^a^	14 (45)
Bisexual	—	6 (32)
Straight	—	6 (19)
Hispanic or Latino/a/x	4 (31)	10 (32)
Race, n (%)		
Black/African American	2 (15)	5 (16)
White	6 (46)	16 (52)
Asian	2 (15)	3 (10)
Hispanic/Latino only	3 (23)	6 (19)
American Indian/Alaskan Native	0 (0)	1 (3)
Highest education, n (%)		
Never graduated high school	—	1 (3)
High school or GED^b^	—	14 (45)
Some college	—	11 (35)
College graduate	—	4 (13)
Any postgraduate studies	—	1 (3)
Sex partners in past 3 months, median (IQR)	—	3 (2-4)
Has health insurance, n (%)	11 (85)	25 (81)
Private	—	10 (32)
Medicaid/Medical	—	12 (39)
Not sure	—	3 (10)
Has primary care provider, n (%)	9 (69)	17 (55)
Prior knowledge of PrEP^c^, n (%)	—	20 (65)
Prior use of PrEP (not current), n (%)	2 (15)	7 (23)
Started PrEP via PrEPmate, n (%)	—	10 (32)
In process of getting on PrEP via PrEPmate, n (%)	—	14 (45)
Interest in taking PrEP^d^, n (%)		
Very interested	—	12 (39)
Somewhat interested	—	5 (16)
A little interested	—	3 (10)
Not interested at all	—	1 (3)

a Data not available for focus groups.

b GED: General Educational Development.

c PrEP: pre-exposure prophylaxis.

d Among 21 participants who did not already start PrEP at time of survey.

Regarding acceptability, the mean and median SUS scores were 68.8 and 70 (IQR 55‐83) out of 100, falling in the “good” range, and the mean and median Client Satisfaction Scores were 28 and 30 (IQR 25‐31) out of 32, indicating high satisfaction (above 26). Most participants (24/31, 77%) reported that PrEPmate helped them get access to PrEP, 31 (100%) would recommend PrEPmate to a friend, and 29 out of 31 (94%) were mostly to extremely satisfied with PrEPmate.

During the study, there was a mean and median of 5.9 and 3 outcoming messages and 5.3 and 2 incoming messages per client enrolled in PrEPmate. In a thematic analysis of text message conversations, key navigation services provided via PrEPmate included answering questions about PrEP, STIs, doxycycline postexposure prophylaxis, and other health concerns; providing information about the local PrEP clinic and the process for getting on PrEP; answering questions about insurance coverage and cost; assisting patients with getting on a patient assistance program; setting up a time for a phone call to discuss PrEP; scheduling and rescheduling PrEP appointments; and arranging transportation to the clinic. In some cases, navigators assisted clients with accessing PrEP via alternate sources (eg, online PrEP delivery program). For those who started PrEP, PrEPmate messages provided an opportunity to check in about adherence and served as a reminder to take PrEP.

### Exit Interviews

#### Ease of Use and Convenience of Texting

Nine participants completed follow-up exit interviews approximately 1 month after enrollment. Most participants found TakeMeHome and PrEPmate straightforward and easy to use, had no issues with answering questions or learning about PrEP, and found the language easy to understand. One participant in California said,

*I didn’t find it hard. I didn’t have to search too hard for information. It was easily accessible. And it was understandable, as well. It wasn’t something that I had to Google and try to figure out. It was right there, very self-explanatory.*

In general, participants did not have issues being directed to another site, and most individuals did not feel like this was a trick or spam or click bait, although 1 participant wanted confirmation that their kit order had gone through before being redirected to the PrEPmate site. They appreciated being connected to a local lesbian, gay, bisexual, transgender, and queer resource and getting assistance through this agency.

One participant in California said about the messages from PrEPmate,

*Very nice, easy to read, no big words. Me, dumb-dumb, can understand. I also think that it wasn’t, like, flooding my inbox…It wasn’t, like, spam messages. It felt like real information, and it felt like it was written by a person.*

When asked what he liked about PrEPmate, another participant in Texas said,

*Well, I just liked it because it was more of—it was very motherly. It was like checking up on me.*

#### Positive Experiences With the Humanized, Responsive Navigator

Participants found PrEPmate easy to use and helped them with various aspects of getting on PrEP. They especially appreciated being connected to a navigator who was able to answer their questions and set up a PrEP intake appointment. A participant in California said,

*The overall experience of the PrEPmate navigator, I would say it would be like a 10 out of 10. They do explain, and they do break down how it works. And they do what you understand how to use it and what day or time, you can schedule your appointment with them. They can help you. They’re very helpful, too, because they will let you know how to use it and stuff like that.*

Another participant in Texas said,

*I think it’s helped a lot because it helped me book my appointments and helped check up on me. And it lets me ask questions and stuff when I’m curious about how it works.*

Participants found that staff responded to text messages in a timely manner, and also appreciated the frequency of check-ins, which maintained their interest in getting started on PrEP. When asked about the frequency of check-ins, one participant in California remarked,

*Actually, I felt good about it because it helped me not forget. Honestly, if it took weeks for you guys to get back, it would have faded away, and I would have lost interest in the program. So it was perfect actually. It kept me engaged.*

This participant appreciated the ability to get her questions answered and set up an appointment quickly, saying

*They made sure every step of the way, do you have any questions? “Hey, I just wanted to just see how everything is going.” It’s very—what’s the word—consistent, and they really cared… And I was able to do everything straight from my phone. Yeah, I didn’t have to sit in a long line or wait at a long doctor’s office.*

#### Recommendations to Improve Functionality and User Experience

As the overall system usability rating was “good,” participants did have some recommendations for improvements. One participant suggested providing more information about PrEP and where it can be accessed before signing up for PrEPmate. Two participants had concerns about being transferred to a different site before ensuring their testing kit order was confirmed and wanted that task to be completed before being transferred to PrEPmate. While most participants understood that the PrEPmate messages were coming from a real person, 1 participant initially thought the messages were coming from a robot or artificial intelligence. To improve PrEPmate, they suggested,

*I would make it feel more like a human because I didn’t know it was a human, to be honest…I would say probably add a name or an emoji or whatever, something that a robot (wouldn’t) do.*

Another participant in Texas suggested that having an app with additional functionality (including the ability to order PrEP delivery and track shipments) would be helpful. A participant in California found the hours of the PrEP clinic restrictive and recommended that PrEP facilities have available appointments after 5 PM or on weekends. Finally, 1 participant in California thought it would be helpful for the system to gather some information about insurance to provide better insurance navigation. She said,

*If there was a feature that was like, “Okay, if you tap on it, you’d be able to link your medical insurance information…” If I could find my insurance company, and then, boom, log in or create an account. And then, boom, you guys have all my insurance information.*

## Discussion

### Principal Findings

This study describes findings from formative focus groups and a pilot study to develop and refine a novel strategy to link home HIV and STI testers using TakeMeHome to PrEP services via an SMS text messaging–based navigation program, PrEPmate. Key findings from the formative phase included having a clear description of PrEPmate services on the TakeMeHome site and stating that PrEP is available at low or no cost; having information about the partnership between TakeMeHome and PrEPmate and confidentiality of the text-messaging system on the PrEPmate landing page; and having weekly or biweekly text message check-ins regarding PrEP navigation using casual, conversational language. The majority of the recommendations from the focus groups were incorporated into the intervention prior to pilot testing. In a 1-month pilot of the finalized linkage and navigation program, nearly one-third of participants enrolled in PrEPmate began using PrEP, and there was high acceptability of this program assessed via survey and exit interviews. Our quantitative assessments included the SUS, which measured ease of use, and the CSQ-8, which measured the helpfulness of and satisfaction with the program.

Home HIV and STI testing offers a number of benefits, including increased privacy, convenience, and the ability to reach populations who have not previously tested [24-26], and integrating HIV and STI self-testing with low-barrier HIV prevention services can help close the “testing-to-prevention” gap. While a number of online HIV and STI testing programs designed to support HIV and STI testing have demonstrated increases in testing rates [5,10,27], these programs either do not have information about PrEP or only passively provide information about these prevention services. In the Adolescent Trials Network studies of 2 mobile apps designed to support HIV testing and PrEP uptake, the MyChoices and LYNX apps demonstrated an increase in HIV and STI testing rates, but no significant increase in PrEP uptake, suggesting that different modalities and a more active approach may be needed to successfully link home HIV and STI testers to PrEP services [28]. While mobile apps require downloading from an app store, TakeMeHome is a website available via one click from a dating app, reducing the initial barrier to engagement. Additionally, the text messaging component of PrEPmate offers an interactive method of weekly follow-up via text message to facilitate ongoing navigation and linkage to a PrEP provider.

A number of text-messaging interventions have been shown to increase PrEP retention in care and adherence [29]. The individualized Texting for Adherence Building intervention increased the likelihood of having near-perfect adherence among stimulant-using men who have sex with men (MSM) in the United States [30], and the PrEPmate intervention increased PrEP retention in care and adherence among young MSM [15]. This study extends the use of text-messaging to support PrEP linkage and navigation, earlier steps in the PrEP continuum. Text messaging interventions are inexpensive, widely available, and scalable, and they offer the ability to provide timely reminders and check-ins, deliver personalized messaging and support, and address stigma by using affirming language and offering a private way to receive support and information without going into a clinic [31-33]. One of our key findings is the usefulness of providing remote access to a PrEP navigator via PrEPmate. PrEP navigators can provide PrEP education and support, assist with insurance and medication access, connect clients with PrEP providers, and facilitate appointment scheduling and reminders, and several studies have demonstrated the positive impact of PrEP navigation on PrEP uptake [34-36]. In the THRIVE PrEP demonstration project, MSM who used navigation were 17 times more likely to link to PrEP compared with MSM who did not use navigation [35]. Additionally, a brief patient navigation intervention increased PrEP prescriptions, PrEP retention in care, and persistence among Black MSM in the Southern United States [33]. In our study, participants found the navigator to be especially helpful in answering their questions, getting a PrEP appointment scheduled, addressing cost and coverage issues, and following up to ensure successful linkage to the clinic. Nearly half of participants enrolled in PrEPmate who completed the survey were African American or Latinx, and 10% were transgender, which reflected the demographics of TakeMeHome testers in these jurisdictions. This model was able to effectively reach populations disproportionately affected by HIV.

Several digital health interventions have recently been tested to increase PrEP uptake and can serve as a benchmark for this study. In the HealthMindr PrEP study of a mobile HIV prevention smartphone app among MSM, 24% of eligible screening participants enrolled in the study, and among those assigned to the intervention arm, 32% reported starting PrEP over a 12-month follow-up period [37]. Similarly, in a randomized controlled trial of the Mobile Messaging for Men (M-cubed) app in MSM, 40% of eligible screening participants enrolled in the study, and among those in the intervention arm, 19% started PrEP [38]. In our pilot study, 16% of TakeMeHome testers who reported an HIV-negative HIV status enrolled in PrEPmate, and of those, 30% were in the process of starting PrEP. While our pilot study had a shorter follow-up duration and was not a fully powered randomized controlled trial, our preliminary data suggest that enrollment and PrEP uptake rates for PrEPmate are within a similar range to other recent trials of digital PrEP linkage interventions.

The positive findings from this pilot study support additional evaluation of the PrEPmate navigation program across a larger number of EHE jurisdictions currently using TakeMeHome and more diverse populations to fully evaluate the effectiveness of this intervention on PrEP uptake and persistence across a range of settings. While local PrEP navigators were used in this pilot study, future studies could investigate the use of a central PrEP navigator to provide support across multiple sites. This approach could be useful in locations without PrEP navigators available and increase efficiency and scalability of the intervention, although information on local PrEP clinics and providers will need to be collected and updated on a regular basis, and insurance navigation and scheduling clients for new PrEP intake appointments may be more challenging when done via a central navigator. Future studies should also evaluate the potential for integrating this intervention into existing public health infrastructure, such as local health departments or community-based organizations and clinics, as well as the cost and cost-effectiveness of different models of implementation. Resources needed to sustain SMS text messaging navigation, including staffing (eg, local or central PrEP navigators), training materials and staff, and the secure interactive SMS text messaging platform supporting PrEPmate, can be further explored in these studies. As a number of health departments and local jurisdictions are already implementing TakeMeHome and have PrEP navigators on staff, this workforce could be leveraged to support the integration of PrEPmate into existing programs. Finally, future studies could investigate the use of automated analytics and artificial intelligence or machine learning to identify individuals testing through TakeMeHome and most in need of navigation support.

This study had several limitations. First, the number of participants enrolled in the focus groups and pilot study was small and consisted of nonrepresentative convenience samples, and therefore, it was not designed to evaluate the efficacy of the intervention. Also, due to the short duration of the pilot, the final PrEP status of some participants was not available, and we were not able to evaluate PrEP persistence for those who initiated PrEP, as other efficacy studies have followed all participants for 12 months. Another limitation is that the demographics and analytics available from TakeMeHome were incomplete, and we were unable to assess how many participants completed the PrEP questions on the TakeMeHome website and made it to the final page prior to being directed to the PrEPmate landing page; these data would have allowed comparisons between individuals enrolling in PrEPmate versus those who did not and provided more granularity into the drop-off at each stage and whether additional revisions to the TakeMeHome PrEP pages would be useful. Social desirability may have impacted participant responses in the focus groups and technical pilot; however, we used computer-assisted self-interview to assess usability and acceptability, paradata metrics from TakeMeHome and PrEPmate to objectively assess feasibility, and participants in focus groups and exit interviews were encouraged to provide honest feedback that would help us best improve the linkage intervention. Due to the pilot nature of this study, we did not collect data on time spent by navigators or the cost and cost-effectiveness of the intervention; it is recommended that future studies assess these implementation outcomes. Additionally, there may be potential selection bias among digitally engaged users of TakeMeHome. Finally, for both phases of this study, participants were only enrolled from Sacramento and Tarrant counties, potentially limiting the generalizability of the findings, although both regions are in high priority EHE jurisdictions. Despite these limitations, our study had several strengths, including the user-centered approach incorporating input from our participants into the final design of the intervention, and the mixed methods approach to evaluate feasibility and acceptability in our pilot study.

### Conclusions

Through user-centered design, we developed a novel SMS text messaging PrEP navigation program to link TakeMeHome home HIV and STI testers to local PrEP services. Preliminary testing in a pilot study demonstrated high feasibility and acceptability of the program and substantial uptake of PrEP among clients enrolled in PrEPmate and supports further evaluation of this program across additional EHE sites in the United States.

## Supplementary material

10.2196/83733 Multimedia Appendix 1 Sociodemographic and behavioral characteristics of overall TakeMeHome users vs PrEPmate survey completers in Tarrant County, TX, and Sacramento County, CA.

## Abbreviations

BHOC: Building Healthy Online Communities
CDC: Centers for Disease Control and Prevention
CSQ-8: Client Satisfaction Questionnaire-8
EHE: ending the HIV epidemic
MSM: men who have sex with men
PrEP: pre-exposure prophylaxis
STI: sexually transmitted infection
SUS: System Usability Scale
