# Supplementary material for: Integrating Mobile Text Messaging Pre-Exposure Prophylaxis Navigation Services Into a Home HIV and Sexually Transmitted Infection Self-Testing Program in the United States: Formative Work and Pilot Implementation Study
Source: JMIR Form Res. 2026 Apr 21;10:e83733. doi: 10.2196/83733 (PMC13099030; doi:10.2196/83733)
Supplement: Multimedia Appendix 1 [file formative-v10-e83733-s001.docx]

Multimedia Appendix 1. Sociodemographic and behavioral characteristics of overall TakeMeHome users vs. PrEPmate survey completers in Tarrant County, TX and Sacramento County, CA

|  | Overall Takemehome (n=91) | PrEPmate survey completers (n=31) | Fisher Exact p-value |
| --- | --- | --- | --- |
| Age <30 | 30 (33%) | 11/29 (38%) | 0.66 |
| Black | 13 (14%) | 5 (16%) | 0.78 |
| Latino | 12 (13%) | 6 (19%) | 0.39 |
| Cisgender man | 46 (51%) | 17 (55%) | 0.84 |
| Transgender woman | 2 (2%) | 3 (10%) | 0.10 |
| Cisgender woman | 39 (43%) | 11 (35%) | 0.53 |
| >1 sex partner past year | 57 (63%) | 23 (74%) | 0.28 |
